# Fine mapping and transcriptomic analysis reveal *BnaMMS*, a novel locus regulating multi-main-stem phenotype via auxin pathway in rapeseed

**DOI:** 10.1186/s12870-026-09172-8

**Published:** 2026-06-04

**Authors:** Rui Xia, Jie Liang, Pengfei Wang, Wanli Zhang, Wen Wang, Qing Li, Lianjin Zeng, Xue Li, Haiyan Zhang, Chengcheng Si, Dengfeng Hong, Xianming Zhou

**Affiliations:** 1 School of Breeding and Multiplication (Sanya Institute of Breeding and Multiplication), Hainan University, Sanya, 572025 China; 2 National Key Laboratory of Crop Genetic Improvement, Hubei Hongshan Laboratory, Huazhong Agricultural University, Wuhan, 430070 China; 3 School of Tropical Agriculture and Forestry, Hainan University, Danzhou, 571737 China; 4 Yazhouwan National Laboratory, Sanya, 572025 China

**Keywords:** *Brassica napus*, Multi-main-stem, BSA-seq, *BnaMMS*, Transcriptome, Auxin

## Abstract

The multi-main-stem (MMS) trait presents considerable potential for improving plant architecture and increasing yield in rapeseed (*Brassica napus* L.). However, the molecular mechanism underlying MMS formation remains poorly understood. In this study, we characterized a dominant MMS material, 1030R. Genetic analysis indicated that the MMS phenotype is controlled by a single dominant gene, designated *BnaMMS*. Utilizing BSA-seq combined with fine mapping, *BnaMMS* was localized to a 70 kb physical interval on chromosome A6, representing a novel genetic locus for MMS regulation. This region contains 17 annotated genes. Sequence analysis revealed multiple SNP and Indel variations between the parental lines. Transcriptome profiling further demonstrated that, among these candidates, two genes (*BnaA06G0092000ZS* and *BnaA06G0092200ZS*) were differentially expressed between near-isogenic lines (NILs) displaying MMS and single-main-stem (SMS) phenotypes. Mechanistic investigations suggested that, with no significant changes observed in the expression of core WUS-CLV pathway genes in our transcriptome data, *BnaMMS* likely regulates MMS formation by disrupting auxin biosynthesis and distribution. Our work identifies a novel genetic locus controlling MMS and provides valuable genetic resources and a theoretical framework for elucidating the molecular mechanisms of stem development and for breeding high-yielding rapeseed varieties with optimized plant architecture.

**Supplementary Information:**

The online version contains supplementary material available at 10.1186/s12870-026-09172-8.

## Introduction

Rapeseed (*Brassica napus* L.) is a globally important industrial crop for both edible oil and biofuel production, whose yield is closely tied to its plant architecture [8]. To meet the demands of modern agricultural practices, particularly mechanized harvesting and high-density planting, breeding varieties with ideal plant architecture, such as compact branching and concentrated pod placement, has become a key objective [8, 26, 33]. In cereal crops, increasing productive tiller number is an effective strategy for yield enhancement [28]. Similarly, in rapeseed, inducing a multi-main-stem (MMS) phenotype, where a single plant develops multiple dominant stems, may be a potential approach to overcome the yield limitation of a single stem and improve population yield [14].

The activity and differentiation patterns of the shoot apical meristem (SAM) are central to determining aerial plant architecture [16, 39]. Auxin regulates floral primordia initiation through polar transport [9], and interacts with hormones like cytokinins to influence SAM size [4, 31, 32]. The canonical WUS-CLV feedback loop, in concert with hormonal signals such as cytokinins and auxin, maintains stem cell homeostasis in the SAM and regulates organ initiation [7, 10, 18, 42]. Therefore, normal SAM development underpins single-stem formation, whereas its aberrant activation or differentiation may lead to the MMS phenotype.

In recent years, genetic studies on the MMS trait in rapeseed have begun to emerge. Using linkage analysis, several quantitative trait loci (QTLs) associated with MMS have been identified [15, 37, 40]. For instance, Zhao et al., [40] mapped two environmentally stable MMS QTLs on chromosomes A03 and C03 and proposed several candidate genes. At the molecular level, analyses of natural mutants have revealed that MMS formation is associated with abnormal SAM development, yet the underlying regulatory networks appear diverse. Wang et al., [29] found that the MMS phenotype in the *ms* mutant correlated with altered expression of cytokinin metabolism genes (e.g., upregulation of *LOG8* and downregulation of *CKXs*). Conversely, Zhu et al., [44] observed upregulation of cytokinin biosynthesis genes (e.g., *LOG5*, *LOG6*) and elevated levels of active cytokinins in the *dt* mutant. These findings suggest that, across different genetic backgrounds, MMS may be triggered by distinct initial metabolic or signaling perturbations.

Despite these advances, the key genes and predominant molecular pathways underlying MMS formation in rapeseed remain largely unknown. It also remains to be determined whether additional regulatory modules, including those independent of the cytokinin pathway, are involved. In this study, we identified a rapeseed line, 1030R, exhibiting a stable MMS phenotype. Genetic analysis indicated that this trait is controlled by a single dominant gene. To decipher its genetic basis, we employed an integrated strategy combining bulked segregant analysis sequencing (BSA-seq), fine mapping, and Ribonucleic acid sequencing (RNA-seq). We successfully mapped a novel locus, designated *BnaMMS*, to a 70-kb interval on chromosome A06. Intriguingly, unlike previously reported mutants, transcriptomic profiling suggested that *BnaMMS* might induce the MMS phenotype by disrupting auxin biosynthesis and distribution, rather than through the classical WUS-CLV pathway or cytokinin signaling. This study not only provides a novel and significant genetic locus for rapeseed architecture breeding but also offers fresh insights and genetic resources for elucidating the molecular mechanisms controlling stem development in *Brassica napus*.

## Materials and methods

### Plant materials and population development

Two rapeseed (*Brassica napus* L.) materials 1030R and FM195, which were provided by Rapeseed Laboratory of Huazhong Agricultural University, exhibited MMS and single-main-stem (SMS), respectively, were used as parental lines in this study. To investigate the genetic basis of MMS, several populations were developed: F_1_ plants were generated by crossing 1030R with FM195; F_2_ populations were obtained by self-pollination of F_1_ plants; and BC_1_ populations were produced by backcrossing FM195 plants to F_1_. The field experiment was conducted during the 2020–2023 growing season at Wuhan. To facilitate transcriptomic and mechanistic analyses, a pair of near-isogenic lines (NILs) contrasting in the MMS trait were developed refer to a previous study [43]. Starting from an F_1_ plant (1030R×FM195), three successive backcrosses were performed using FM195 as the recurrent parent, followed by two generations of selfing to obtain BC_3_F_2_ populations. Throughout the backcrossing and selfing generations, foreground selection was carried using flanking markers tightly linked to the *BnaMMS* locus (S42 and S31), while background selection was performed using genome-wide SSR markers to accelerate the recovery of the recurrent parent genome. From the BC_3_F_2_ population, two homozygous lines were selected: one carrying the 1030R allele at the *BnaMMS* locus (NIL-MMS) and the other carrying the FM195 allele (NIL-SMS). These NILs were used for subsequent RNA-seq.

### Field experiment and phenotypic evaluation

Field trials were conducted during the normal rapeseed growing season. Plants were grown in a randomized complete block design with three replicates in Wuhan, China. Each row contained 10–12 plants, with a spacing of 20 cm between plants within the row and 25 cm between rows. Field management followed standard agronomic practices. For phenotypic investigation, the number of main stems per plant was recorded at the bolting stage. A plant was classified as MMS if it developed two or more distinct main stems, and as SMS if only one main stem was present. The SAM morphology of seedlings was examined under a stereomicroscope (NikonSMZ25). For transcriptome sampling, apical meristem tissues were collected from seedlings at the same developmental stage, immediately frozen in liquid nitrogen, and stored at -80 ℃ until RNA extraction.

### BSA-seq and fine mapping of *BnaMMS*

BSA-seq was performed using an F_2_ population. Two bulks were constructed: the MMS bulk (M1F-pool) comprised 25 individuals showing the MMS phenotype, and the SMS bulk (N1F-pool) comprised 25 SMS individuals. Genomic DNA was extracted from young leaves using a Plant Genomic DNA Kit (Tiangen, Beijing, China). Whole-genome resequencing of the two parents and the two bulks was conducted on an Illumina NovaSeq 6000 platform (2 × 150 bp read length). Clean reads were aligned to the *Brassica napus* reference genome ZS11 [25]. SNP and Indel annotations were performed using ANNOVAR [27] with the ZS11 reference genome annotation [25]. Variants were categorized into exonic (further subclassified as synonymous, non‑synonymous, stopgain, or frameshift), intronic, splicing, 5’‑UTR, 3’‑UTR, upstream (within 1 kb of the transcription start site), downstream (within 1 kb of the transcription end site), or intergenic regions based on the RefGene annotation. SNPs and Indels were annotated using ANNOVAR [27]. The SNP index, G’ value and Euclidean distance (ED) algorithms were applied using easyQTLseq (https://github.com/laowang1992/easyQTLseq.git) to identify candidate regions associated with the MMS trait. A 95% confidence interval under the null hypothesis of no QTL was used to define the preliminary mapping interval.

Based on the BSA-seq results, Indel markers were designed within the candidate region on chromosome A6. Polymorphic markers between the parents and bulks were selected for genotyping a large F_2_ population. Recombinant individuals were identified, and the candidate region was narrowed using additional Indel markers. For further fine mapping, a BC_3_F_2_ population was developed, and the recombinant plants were genotyped with newly developed polymorphic Indel markers. The physical position of markers was determined according to the ZS11 genome assembly. The primers used in this study are presented in Supplementary Table S7.

### RNA-seq and transcriptome analysis

Total RNA was extracted from SAM tissues of two NILs derived from the BC_3_F_2_ population NIL-SMS and NIL-MMS in five-leaf stage. Three biological replicates were prepared for each line. RNA quality was checked using an Agilent 2100 Bioanalyzer. Libraries were constructed with the NEBNext Ultra RNA Library Prep Kit and sequenced on an Illumina NovaSeq 6000 platform. Clean reads were aligned to the ZS11 genome using HISAT2 software [13]. Gene expression levels were quantified as FPKM (Fragments Per Kilobase of exon model per Million mapped fragments) using StringTie (v2.1.5) [19]. Genes with a mean read count across all samples of less than 10 were filtered out prior to differential expression analysis using DESeq2; genes with |log₂FoldChange| > 1 and Padj ≤ 0.05 were considered differentially expressed. KEGG pathway enrichment analyses were conducted using clusterProfiler [35]. GO enrichment analysis of the DEGs was performed using GOseq, with GO terms having a Padj ≤ 0.05 considered significantly enriched [34].

### Statistical analysis

Statistical analysis was conducted using GraphPad Prism 8. Data are presented as mean ± standard deviation. For comparisons between two groups, a two-tailed Student’s *t*-test was applied. A *P*-value of less than 0.01 was considered statistically significant in all tests. Each experiment was performed with three independent biological replicates. The goodness-of-fit of the phenotypic data was assessed using a chi-square (χ²) test in SPSS 16.0 (SPSS Inc., Chicago, IL, USA) to evaluate deviations between the observed values and the theoretical segregation ratios.

## Results

### Phenotypic description and genetic analysis of multi-main-stem

**Fig. 1:**
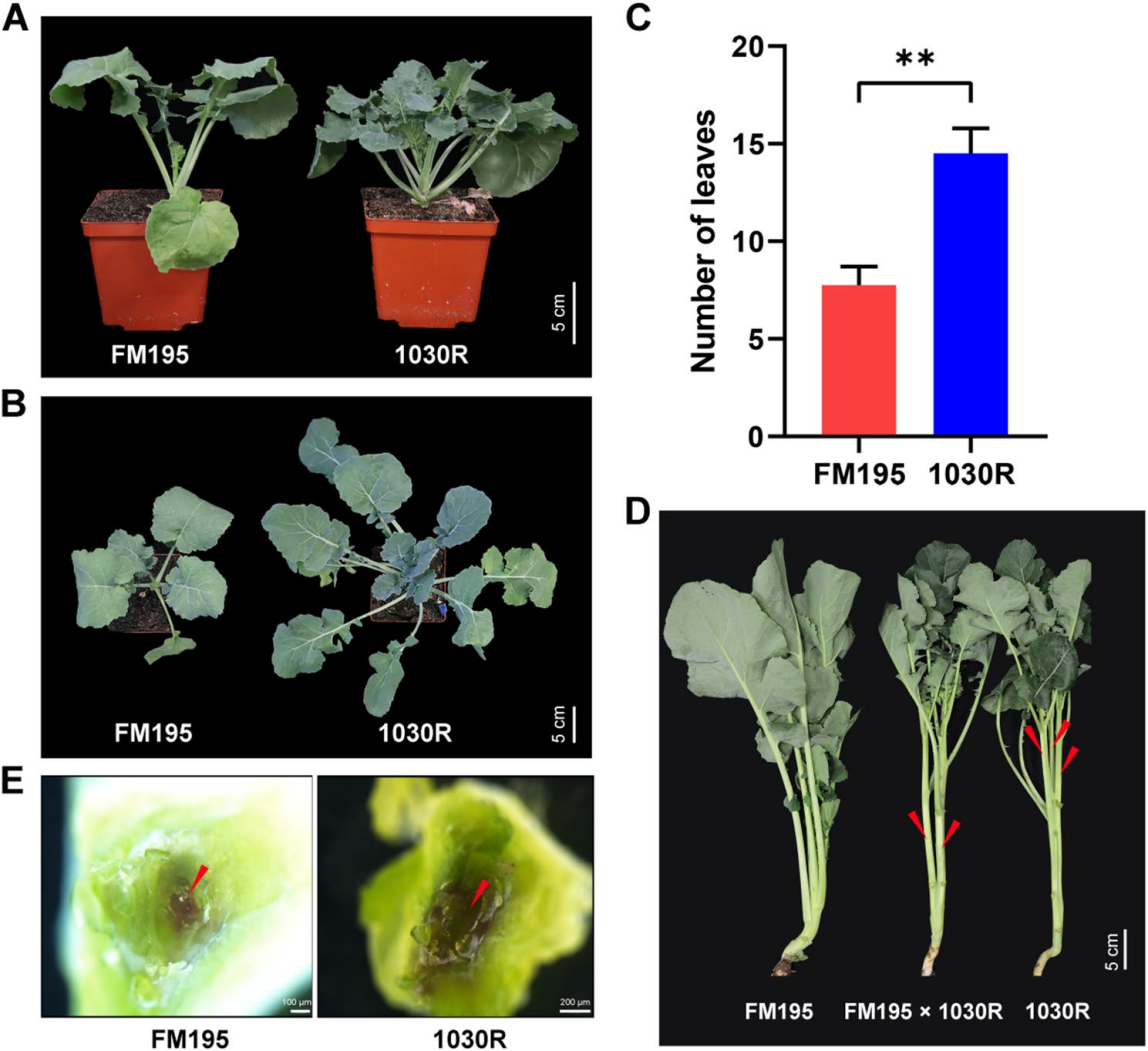
Phenotypes characteristics of multi-main-stem material. Plan view **A** and top views **B** of varieties FM195 and 1030R at the seedling stage. **C** Comparison analysis of leaf number between FM195 and 1030R, assessed at the three-week-old seedling stage after sowing. **D** Phenotypes of FM195, 1030R, and their progenies at the bolting stage. Red arrow indicates the main stem. **E** Stem meristems of FM195 and 1030R at the initial stage of differentiation. Red arrow indicates the shoot apical meristem

1030R is an MMS material derived from the breeding process, and it maintains stable inheritance. Compared to the SMS parent FM195, the seedlings of 1030R exhibited more leaves during the vegetative development stage (Fig. 1A-C). In addition, multiple growth points can be clearly observed at the seedling stage of 1030R (Fig. 1B). After bolting, unlike FM195 which produces only one main stem, 1030R develops several distinct main stems (Fig. 1D). Similar to 1030R, the hybrid progeny of FM195 and 1030R also display the MMS phenotype (Fig. 1D). To further investigate the origin of MMS, the shoot apical meristems (SAM) of 1030R and FM195 were examined under a stereomicroscope. During the apical meristem formation stage, FM195 developed a single, well-defined growth point with minimal leaf primordia. In contrast, 1030R formed an elliptical meristem that lacked a clear focus and was surrounded by numerous leaf primordia. (Fig. 1E).

To determine the inheritance patterns of MMS, the main stem number of multiple genetic progeny populations derived from 1030R and FM195 was examined. All F_1_ plants exhibited MMS phenotype (Table 1), suggesting that MMS is inherited in a dominant manner. Two F_2_ populations grown in Wuhan were used to investigate the segregation ratio of stem number. According to the results, both populations (F_2_-1 and F_2_-2) showed an MMS/SMS ratio of 3:1 based on Chi-square tests (χ^2^ = 0.096 and χ^2^ = 0.063, respectively). In a BC₁ population generated by backcrossing the recessive parent FM195 to an F_1_ plant, 174 plants showed MMS and 168 showed SMS, fitting a 1:1 segregation ratio (χ^2^ = 0.073). These results indicate that the MMS phenotype is controlled by a single dominant gene, which is designated as *BnaMMS*.

**Table 1:** Segregation analysis of the MMS trait in genetic populations

Population	Total plants	Multi-main-stem plants	Single-main-stem plants	Expected ratio	χ^2^ value‌
F_1_	15	15	0	-	-
F_2_-1	419	312	107	3:1	0.096
F_2_-2	338	252	86	3:1	0.063
BC_1_	342	174	168	1:1	0.073

Chi-square test, χ^2^_0.05, 1_ = 3.84

F_1_, hybrid progeny from the cross 1030R × FM195; F_2_, progeny from self-pollinated F_1_ plants; F_2_-1 and F_2_-2 denote two independent biological replicates of the F_2_ population; BC_1_, progeny from the backcross (F_1_ × FM195)

### Preliminary mapping of *BnaMMS* by BSA-seq

**Fig. 2:**
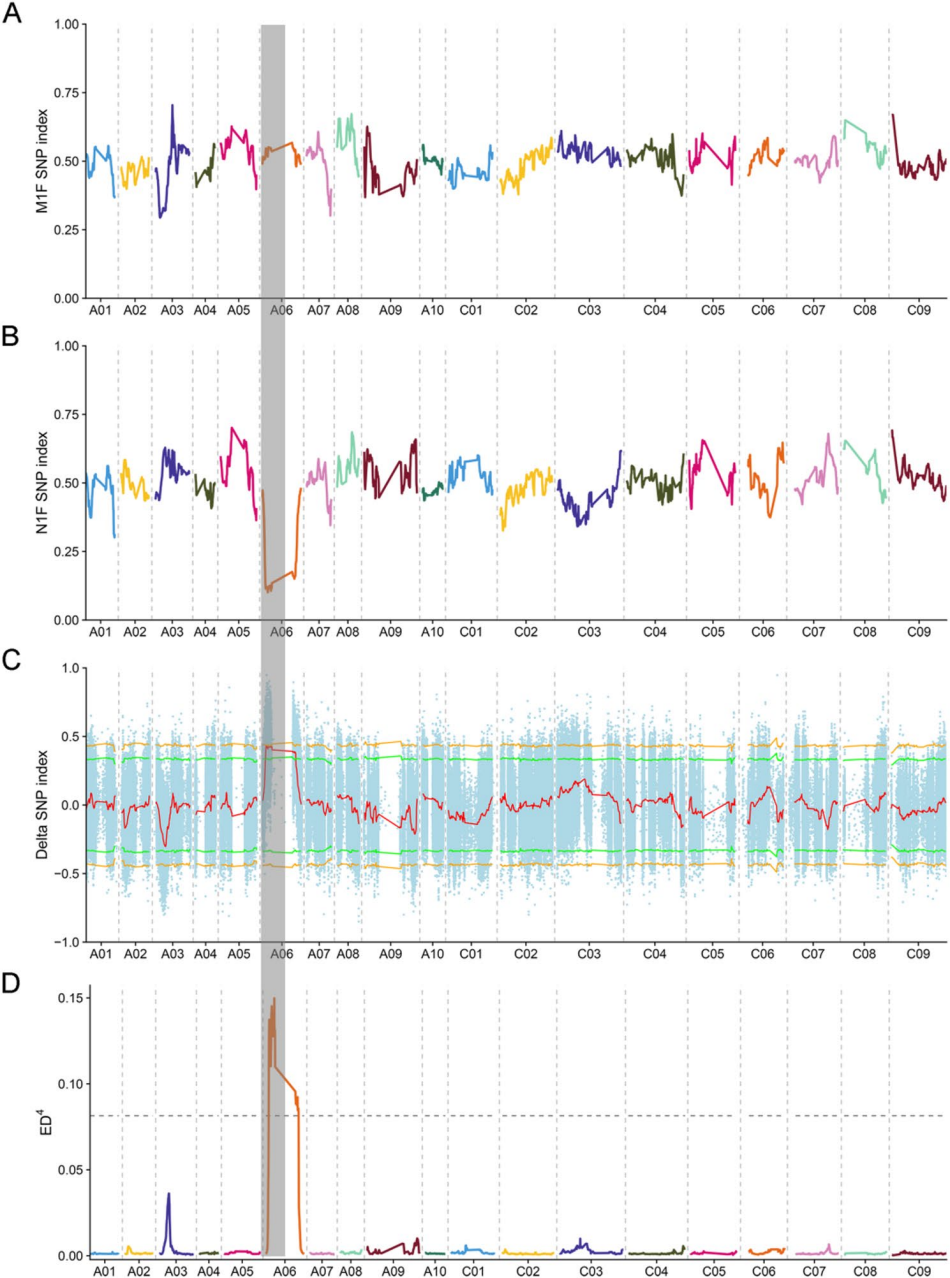
Identification of the *BnaMMS *locus. **A** SNP-index for the MMS bulk (M1F-pool). **B** SNP-index for the SMS bulk (N1F-pool). **C** Delta SNP index plot calculated from the difference between the two bulks. The orange and green horizontal lines represent the 95% (*p* < 0.05) and 99% (*p* < 0.01) confidence intervals, respectively, The 99% CI (7.4 Mb) was used for initial mapping, while the 95% CI is provided for reference, under the null hypothesis of no QTL. **D** Euclidean Distance (ED) values plotted across the genome. The x-axis indicates the physical position on the chromosome, and the y-axis shows the SNP-index (**A**, **B**, **C**) or ED value **D**

To map the *BnaMMS* locus, BSA-seq was performed on an F_2_ population. Whole-genome resequencing was employed to analyze the MMS bulk (M1F-pool), the SMS bulk (N1F-pool), and the two parents. Using the *Brassica napus* ZS11(V0) genome as a reference, the mapping rate of clean reads exceeded 93% (Table S1), with genome coverage > 90% (Table S2). A total of 1,322,615 SNPs were detected between the two pools, and 61,879 of them were distributed on Chromosome A6 (Table S3). The delta SNP index, calculated from the difference in SNP indices between the N1F-pool and M1F-pool, revealed a significant peak at the front end of chromosome A6 (Fig. 2A-C). Using the ED (Euclidean distance) algorithm, a QTL region was also detected in a similar location on chromosome A6, further validating this candidate interval (Fig. 2D).

To further determine the interval of QTL regulating MMS, three algorithms were introduced to analyze SNP variations on chromosome A6. The results showed that these three algorithms (SNP-index, ED, and G’ value) co-detected a common interval (2.4–12.4 Mb) spanning 10 Mb on chromosome A6 (Fig. S1). Within this region, scatter plots from different algorithms exhibited similar distribution patterns (Fig. S1D). Based on a 99% confidence interval (CI), the QTL was initially mapped to a 7.4 Mb region (3.6–11.0 Mb), which was therefore designated as the candidate region for *BnaMMS*.

### Fine mapping of *BnaMMS*

**Fig. 3:**
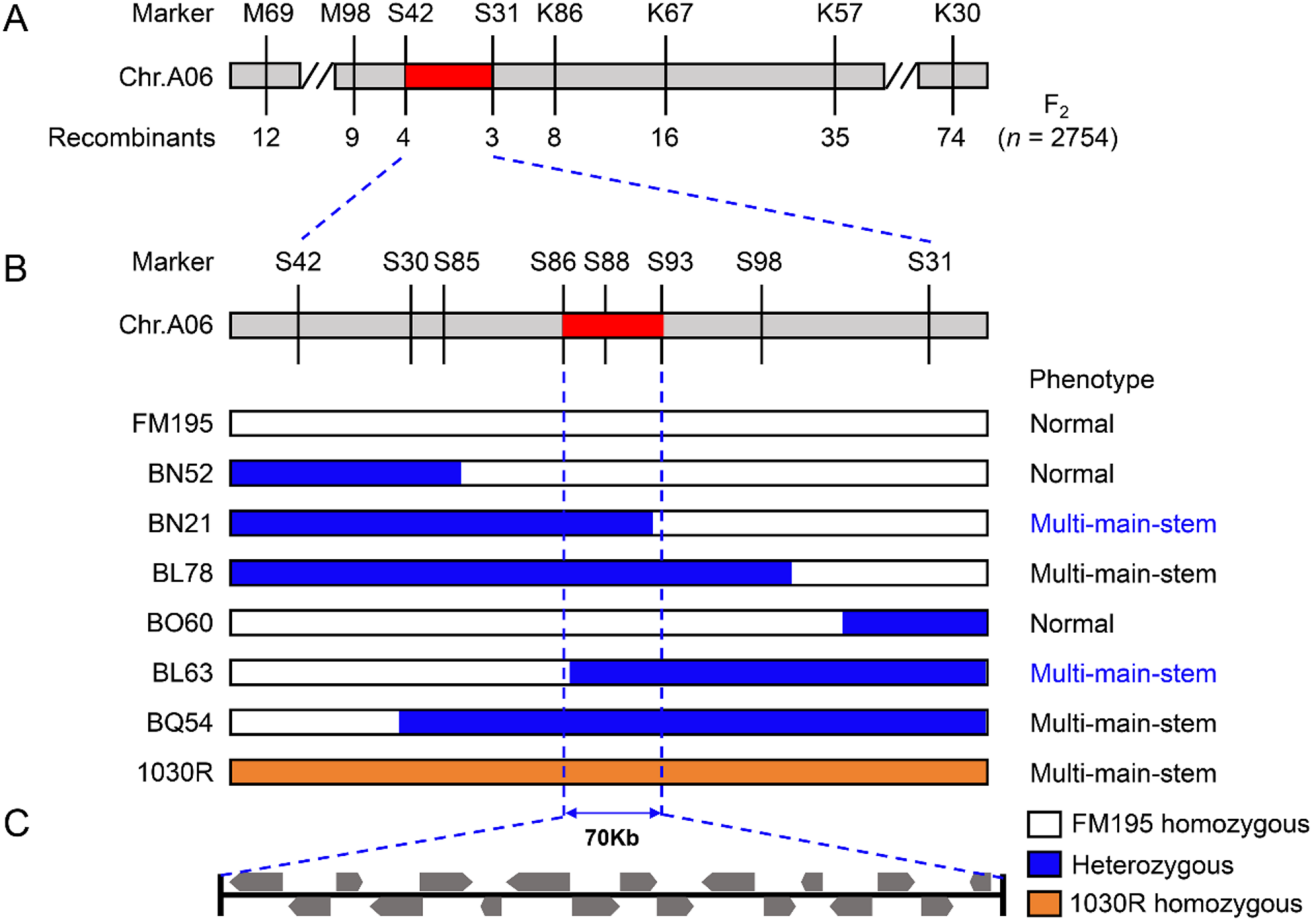
Fine mapping of *BnaMMS*. **A** *BnaMMS* was mapped between S42 and S31 using an F_2_ population. The numbers below the chromosome schematic indicate the count of recombinant individuals between adjacent markers. **B** Fine mapping of *BnaMMS* based on recombinants selected from BC_3_F_2_ population. **C** Annotated genes in the candidate interval. The annotated genes and their transcriptional orientations are represented by gray arrows (indicating direction) and rectangles (indicating exons)

For fine mapping, 34 Indel markers within the candidate region were developed and screened for polymorphism between the parents (1030R and FM195) and the two bulks (M1F-pool and N1F-pool) (Fig. S2A). Eight uniformly distributed polymorphic markers were subsequently used for genotyping an F_2_ population comprising 2754 individuals. *BnaMMS* was located to a 1.2 Mb region between markers S31 and S42 (Fig. 3A). To further narrow down the region of *BnaMMS*, six additional polymorphic Indel markers were developed within the candidate region (Fig. S2B) and used to genotype 373 recombinant individuals selected from a BC₃ population. Based on the genotype and phenotype of these recombinants, *BnaMMS* was delimited to a region between markers S86 and S93, corresponding to a 70-kb physical interval on the ZS11 genome (Fig. 3B).

### Candidate gene analysis

**Fig. 4:**
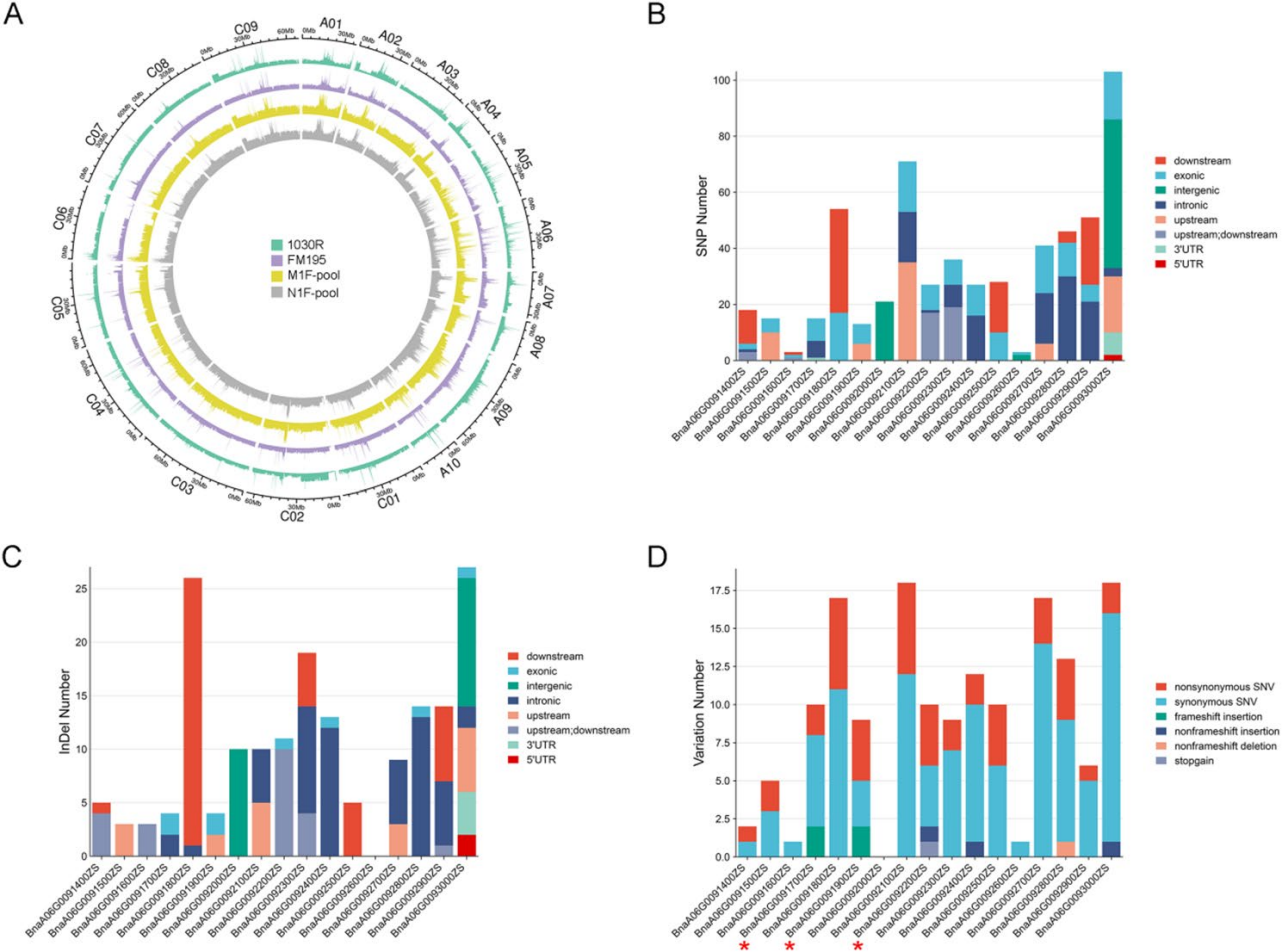
Variation analysis within the candidate region of the parental lines. **A** Sequencing depth and genome coverage achieved for parental resequencing data. **B**, **C** Distribution profiles of SNP **B** and Indels **C** across the gene structures of the 17 candidate genes. **D** Summary statistics of sequence variations identified in each candidate gene. Genes containing frameshift or premature termination mutations are highlighted with red asterisks

Within the 70-kb candidate region, 17 genes were identified and 16 of them were annotated in *Arabidopsis* (Fig. 3C; Table 2). Among these genes, two genes *BnaA06G0091400ZS* and *BnaA06G0091500ZS*, encode IAA oxidase homologs with high similarity to *AT1G14120 (DAO2)* and *AT1G14130 (DAO1)*, respectively. These two genes are known to affect plant growth and development by regulating the homeostasis of auxin [11, 21]. To further analyze the gene sequences within the candidate region, we performed resequencing analysis on both parents. According to the results, the mapped rate and coverage of both parents reached 99% and 90%, respectively (Table S1 and S2, Fig. 4A), with an average sequencing depth > 15× (Fig. S3), indicating high-quality data suitable for mutation detection.

**Table 2:** Annotated genes in the candidate region

BnaA06G0091400ZS	AT1G14120	IAA oxidase
BnaA06G0091500ZS	AT1G14130	IAA oxidase
BnaA06G0091600ZS	AT1G14150	Photosynthetic NDH subcomplex L2
BnaA06G0091700ZS	AT1G14170	RNA-binding KH domain-containing protein
BnaA06G0091800ZS	AT1G14180	RING/U-box superfamily protein
BnaA06G0091900ZS	AT1G14200	RING/U-box superfamily protein
BnaA06G0092000ZS^*^	-	-
BnaA06G0092100ZS	AT1G14230	GDA1/CD39 nucleoside phosphatase family protein
BnaA06G0092200ZS	AT1G14250	GDA1/CD39 nucleoside phosphatase family protein
BnaA06G0092300ZS	AT1G14260	RING/FYVE/PHD zinc finger superfamily protein
BnaA06G0092400ZS	AT1G14270	CAAX amino terminal protease family protein
BnaA06G0092500ZS	AT1G14280	Phytochrome Kinase Substrate 2
BnaA06G0092600ZS	AT1G14290	Sphinganine C (4)-monooxygenase 2
BnaA06G0092700ZS	AT1G14300	ARM repeat superfamily protein
BnaA06G0092800ZS	AT1G14310	Haloacid dehalogenase-like hydrolase (HAD) superfamily protein
BnaA06G0092900ZS	AT1G14320	Ribosomal protein L10
BnaA06G0093000ZS	AT1G14330	Galactose oxidase/kelch repeat superfamily protein

* No clear ortholog found in Arabidopsis; may represent a rapeseed-specific gene

Comparison of the parental resequencing data yielded numerous SNPs and Indels. SNPs were unevenly distributed across chromosomes, predominantly near chromosome ends (Fig. S4). Further analysis revealed that SNPs were mainly located in intergenic regions and introns, with fewer in exons (Fig. S5A and Table S5). The genomic distribution of Indels was similar (Fig. S5B). Most SNPs resulted in synonymous or non-synonymous mutations, with a minority introducing premature stop codons (Fig. S5C and Table S4). For Indels, the most common mutations are frameshift Indels, followed by non-frameshift mutations and then premature termination events (Fig. S5D).

Analysis of the sequences within the candidate regions revealed that all candidate genes between the parents harbored SNP or Indel variations (Fig. 4B, C and Table S6). SNPs were generally more abundant than Indels. While SNPs were detected in the exons of nearly all candidate genes, only a few genes contained exonic Indels. Further characterization showed that most SNPs were synonymous, and the majority of Indels did not cause frameshift mutations. Overall, among the 17 genes examined, 14 carried non-synonymous mutations (Fig. 4D). Among these, two genes (*BnaA06G0091700ZS* and *BnaA06G0091900ZS*) contained frameshift mutations, and one (*BnaA06G0092200ZS*) carried a premature termination codon. Additionally, the two IAA oxidase homolog encoding genes, *BnaA06G0091400ZS* and *BnaA06G0091500ZS*, were also found to harbor multiple exonic SNPs resulting in non-synonymous mutations, which may potentially affect their functions.

### RNA-seq analysis

**Fig. 5:**
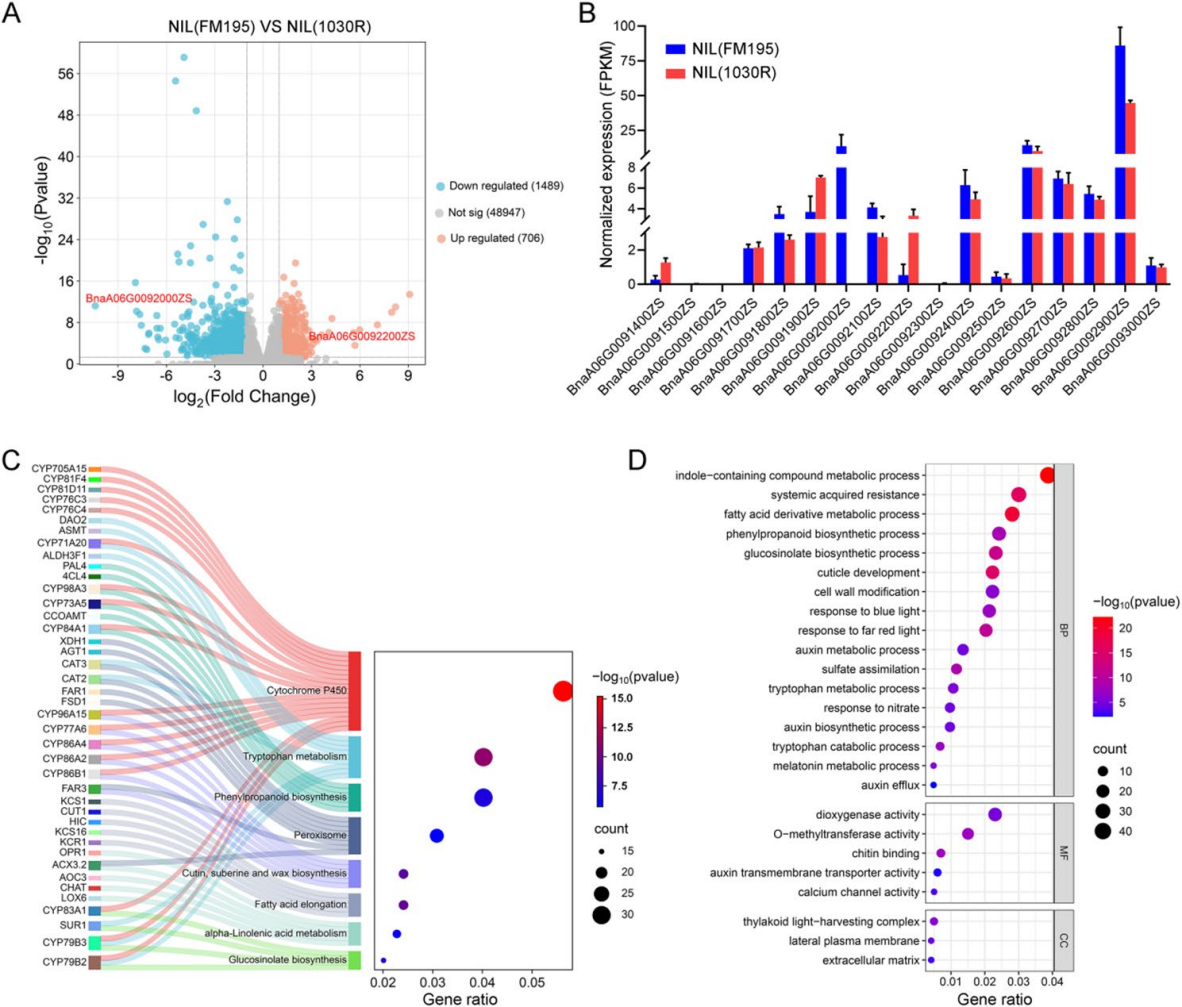
Transcriptome analysis and candidate gene screening. **A** Volcano plot illustrates the distribution of DEGs between the SMS NIL (FM195 background) and the MMS NIL (1030R background). Significantly upregulated and downregulated DEGs are highlighted in pink and cyan-blue, respectively. **B** Expression levels of the 17 candidate genes within the fine-mapped interval, as determined by RNA-seq. **C** Significantly enriched KEGG pathways among the DEGs. **D** Gene Ontology (GO) enrichment analysis for the DEGs, categorized into Biological Process (BP), Molecular Function (MF), and Cellular Component (CC) terms

To further evaluate candidate genes and explore the molecular basis of MMS, the apical meristem tissues of two NILs from the BC_3_F_2_ population, exhibiting SMS and MMS respectively, were subjected to transcriptome analysis. A total of 2195 differentially expressed genes (DEGs) were identified, with 1489 downregulated and 706 upregulated in MMS NIL compared to the SMS NIL (Fig. 5A, and Fig. S6). Notably, three genes (*BnaA06G0091500ZS*, *BnaA06G0091600ZS*, and *BnaA06G0092300ZS*) in the candidate region showed extremely low expression (FPKM < 1) (Fig. 5B). Of the remaining 14 genes, only two (*BnaA06G0092000ZS* and *BnaA06G0092200ZS*) were differentially expressed. The expression level of *BnaA06G0092200ZS* was significantly higher in NIL(1030R) than in NIL(FM195). Interestingly, *BnaA06G0092000ZS*, which was highly expressed in NIL(FM195), was almost not expressed in NIL(1030R).

KEGG pathway analysis of the DEGs showed enrichment in eight pathways, including tryptophan metabolism, phenylpropanoid biosynthesis, and glucosinolate biosynthesis (Fig. 5C). Many DEGs encoding cytochrome P450 enzymes were enriched in these pathways. Subsequently, GO enrichment analysis was conducted, and DEGs were categorized into three groups: molecular function (MF), biological process (BP), and cellular component (CC). Many DEGs were enriched in GO terms related to secondary metabolite synthesis and metabolism, as well as auxin metabolism and transport, including indole-containing compound metabolic process, phenylpropanoid derivative metabolic process, glucosinolate biosynthesis process, auxin biosynthesis process, auxin metabolic process, auxin efflux, and auxin transmembrane transporter activity (Fig. 5D). These results imply that secondary metabolite and auxin synthesis and metabolism are likely involved in regulating MMS formation in rapeseed. Integrated analyses indicate that *BnaA06G0091400ZS* and *BnaA06G0091500ZS* are the most likely candidate genes for *BnaMMS* within the mapped interval. Nevertheless, functional validation via CRISPR or complementation assays is required to confirm their roles.

### Prediction of the MMS formation mechanism

**Fig. 6:**
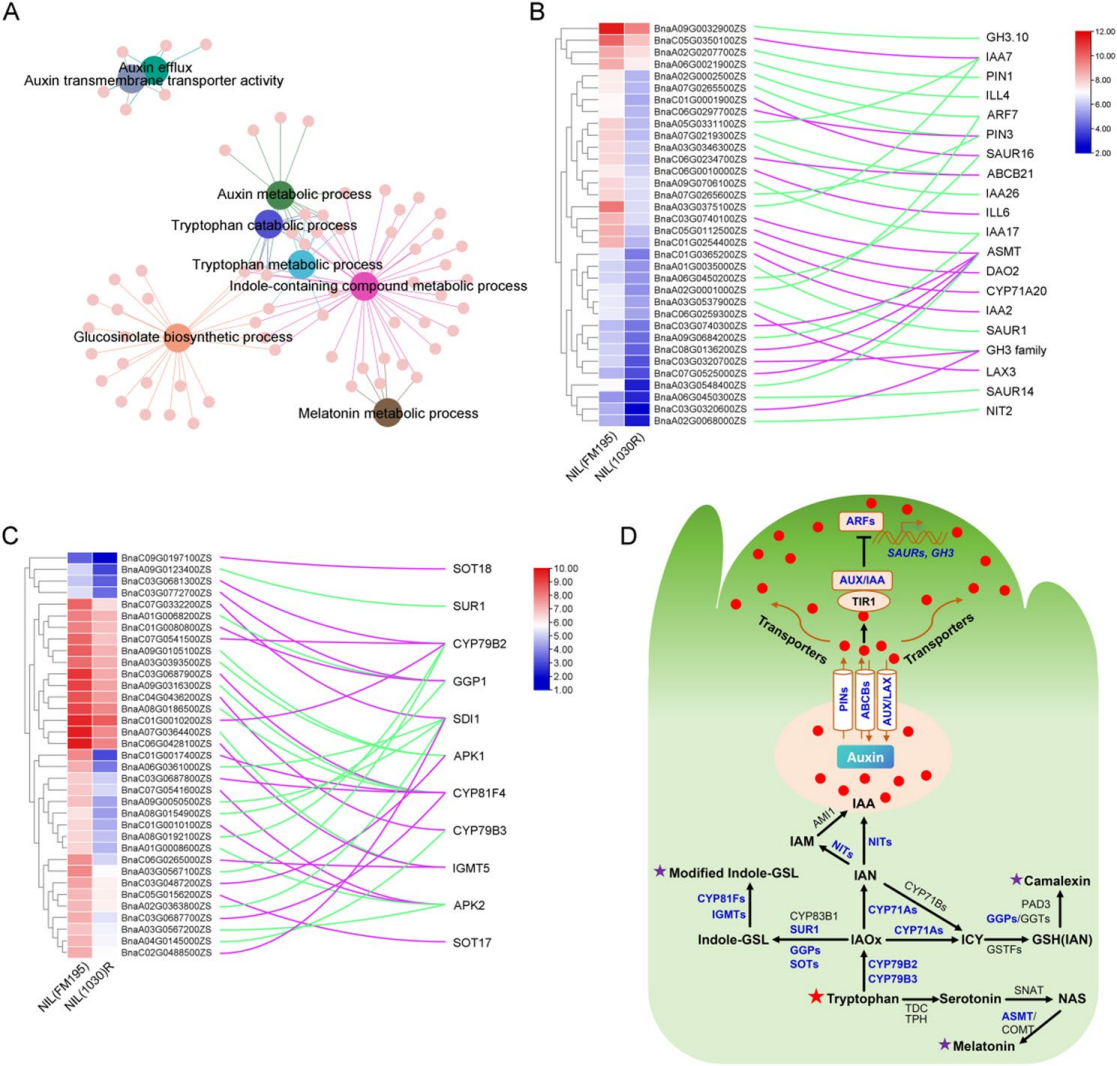
Integrated analysis of DEGs reveals a potential regulatory network for MMS formation. **A** Association network depicting the relationship between significantly enriched biological pathways and the core set of DEGs. Pink nodes represent individual DEGs, while distinctly colored circular nodes denote different metabolic or signaling pathways. **B**, **C** Heatmaps displaying the expression patterns of DEGs implicated in auxin biosynthesis and transport **B** and in tryptophan metabolism **C** between the SMS and MMS NILs. Purple lines represent genes derived from the C subgenome, and green lines represent genes derived from the A subgenome of allotetraploid rapeseed. **D** Proposed model illustrating the mechanism by which *BnaMMS* may regulate MMS development. The model posits that perturbation of tryptophan metabolism (indicated by the purple asterisk) leads to disrupted auxin (IAA, red dots) homeostasis and signaling, ultimately causing SAM abnormalities. DEGs are indicated in blue font

To further elucidate the molecular mechanisms underlying the formation of MMS, we analyzed DEGs enriched in relevant GO terms. The results revealed that a significant number of DEGs are simultaneously involved in multiple interconnected biological processes, including tryptophan metabolic process, melatonin metabolic process, glucosinolate biosynthesis process, auxin metabolism, as well as auxin influx and transmembrane transporter activity (Fig. 6A). Notably, these metabolic pathways are closely linked at the regulatory level.

Expression pattern analysis showed that the majority of identified DEGs were downregulated in 1030R (Fig. 6B, C), with only a few genes related to auxin metabolic process being upregulated (Fig. S7). Furthermore, multiple copies of genes involved in both auxin and tryptophan metabolic pathways were consistently downregulated in 1030R, including *IAA7*, *PIN3*, *ASMT*, *IAA17*, *SAUR16*, *CYP79B2*, *CYP81F4*, and *IGMT5* (Fig. 6B, C).

Integrating the differential expression profiles with associated metabolic pathways, we proposed a model for the regulatory network potentially governing MMS formation (Fig. 6D). The model suggests that in 1030R, the candidate gene *BnaMMS* may perturb tryptophan metabolism, thereby suppressing the expression of genes involved in auxin biosynthesis. This disturbance further extends to downstream branches of tryptophan metabolism, affecting glucosinolate, melatonin, and camalexin pathways. Consequently, disrupted auxin synthesis impairs both auxin transport and downstream signaling responses, leading to an uneven distribution of auxin in the SAM. This imbalance likely results in abnormal expression of genes related to growth and development, ultimately causing aberrant main stem development and the MMS phenotype.

## Discussion

Rapeseed (*Brassica napus* L.) is a major global oilseed crop, with its yield determined by the synergistic effects of factors such as silique number, seeds per silique, and seed weight [24, 43]. Traditionally, a single rapeseed plant produces only one main stem carrying a solitary primary inflorescence, with the siliques on the primary inflorescence accounting for approximately 20%–30% of the total silique number. Previous studies have revealed that the number of siliques on the primary inflorescence is a major yield determinant [5]. In this study, 1030R exhibited a unique MMS phenotype. This trait was governed by a single dominant gene, allowing its efficient transmission to offspring through crossbreeding, thereby offering a straightforward genetic pathway for high-yield breeding. The MMS trait directly increased the number of main stems per plant, significantly enhancing the effective silique number, which represented a direct and effective strategy for boosting individual plant yield.

Currently, rapeseed breeding is transitioning towards mechanization and high-density planting, imposing specific requirements on the ideal plant architecture [12, 38]. These include moderately reduced plant height for improved lodging resistance, compact branching to accommodate high-density planting, and uniform maturity to facilitate mechanized combined harvesting [6, 20, 41]. The MMS trait holds multiple potential benefits for plant architecture improvement and yield enhancement: (1) Promotion of compact architecture: multiple stems may alter apical dominance distribution, optimizing branch angles and spatial configuration. This favors the development of a relatively upright growth habit, improving canopy light use efficiency and the efficiency of mechanized operations. (2) Enhancement of stem lodging resistance: MMS materials are often associated with thicker basal stems and well-developed mechanical tissues, contributing to enhanced population stability under dense planting conditions and adaptability to mechanized harvesting. (3) Coordination of “source-sink” relationship: the stem is a highly efficient “sink” organ. The presence of multiple stems may drive the optimized allocation of photosynthetic assimilates, thereby improving overall population photosynthetic efficiency and yield potential. In a word, the MMS material 1030R, characterized by multiple apical meristem development, may provide a valuable novel germplasm resource and a genetic improvement approach for breaking through the current yield plateau and designing high-yielding ideal plant types suited to modern production systems.

To date, research on the MMS trait in rapeseed remains limited, with most studies focusing primarily on phenotypic description and the identification of genetic relationships [29, 40, 44]. In a previous study, several quantitative trait loci (QTLs) regulating MMS were identified using a doubled haploid (DH) population, among which two QTLs located on chromosomes A3 and C3 were reported to be environmentally stable [40]. In present study, we identified a dominant locus controlling MMS formation in rapeseed. Genetic analysis confirmed that MMS is inherited as a monogenic dominant trait, consistent with segregation patterns in F_2_ and backcross populations (Table 1). Additionally, we identified a novel locus on chromosome A6 through BSA-seq, expanding the genetic landscape governing stem architecture in rapeseed. Then, we delimited *BnaMMS* to a 70-kb region containing 17 annotated genes (Fig. 3). This region differs genomically from the previously reported loci, suggesting that distinct genetic pathways may underlie MMS in different genetic backgrounds. Within this interval, two genes encoding IAA oxidase homologs (*BnaA06G0091400ZS* and *BnaA06G0091500ZS*) were identified as promising candidate genes, based on their established function in regulating auxin homeostasis, a key mechanism governing meristem activity and stem development [17, 36]. *BnaA06G0091400ZS* and *BnaA06G0091500ZS* harbor multiple exonic SNPs between MMS and WT lines. However, RNA-seq analysis showed that their expression levels were not significantly different (Fig. 5B). Thus, it may act through altered protein function rather than differential transcript abundance.

Transcriptome profiling of NILs revealed that only two genes in the candidate region, *BnaA06G0092000ZS* and *BnaA06G0092200ZS*, were differentially expressed between MMS and SMS plants (Fig. 5A, B). The pronounced upregulation of *BnaA06G0092200ZS* and near-complete suppression of *BnaA06G0092000ZS* in 1030R suggest that *BnaMMS* may exert its effects through transcriptional modulation, potentially influencing auxin signaling pathways. It is important to note that the differentially expressed genes identified within the candidate interval (*BnaA06G0092000ZS* and *BnaA06G0092200ZS*) do not carry non-synonymous or frameshift mutations in the parental lines, suggesting they are unlikely to be the primary causal variants. Instead, their altered expression may reflect downstream consequences of the regulatory perturbation caused by the true causal gene, which could act via trans-acting factors or epigenetic mechanisms. Therefore, while RNA-seq provides functional insights, integration with genomic variation is essential to prioritize candidate genes. Notably, our expression data showed no significant alterations in the transcript levels of key genes associated with the WUS-CLV pathway, a well-established regulatory module controlling meristem maintenance and stem determinacy in plants [1, 2, 22]. Instead, we observed extensive differential expressions of genes involved in auxin synthesis, transport, and signaling (Fig. 6B). This contrast suggests that the MMS phenotype in 1030R may arise not from disruption of the canonical meristem maintenance pathway, but rather from perturbations in auxin distribution within the SAM. This hypothesis was further supported by KEGG and GO enrichment analyses, which highlighted the significant involvement of specific auxin-related processes in MMS formation, namely tryptophan metabolism, auxin biosynthesis, and transmembrane transport (Fig. 5C, D). The coordinated downregulation of several auxin pathway genes (e.g., *IAA7*, *PIN3*, and *CYP79B2*) in 1030R further suggests that altered auxin distribution may disrupt normal stem patterning, leading to the observed multi-stem phenotype.

Although our transcriptome data did not detect significant differential expressions of key genes involved in CK biosynthesis or metabolism (such as *IPTs*, *LOGs*, or *CKXs*), this does not imply that CK signaling is irrelevant to MMS formation. On the contrary, previous studies strongly suggest that CK is a key regulator of meristem activity [3, 23, 30]. For instance, Wang et al., observed upregulation of the CK synthesis gene *LOG8* and downregulation of several *CKX* genes in the *ms* mutant, proposing that disruption of CK signaling is a major cause of SAM abnormality and the MMS phenotype [29]. Similarly, candidate genes identified by Zhao et al., included components potentially involved in hormone response or interaction [40], while in the *dt* mutant, upregulation of genes such as LOG5 and LOG6 along with increased levels of active cytokinins were observed, suggesting that enhanced CK activity may directly contribute to SAM activation and the MMS phenotype [44]. These findings indicate that in different genetic backgrounds, the MMS phenotype may be triggered by distinct initial metabolic or signaling perturbations. In 1030R, the primary transcriptional disturbance is centered on the auxin pathway, whereas in materials like the *ms* and *dt* mutants, the CK pathway exhibits more pronounced changes. This divergence likely reflects the genetic complexity and pathway redundancy underlying MMS formation in rapeseed.

Nevertheless, several questions remain unresolved. The functional identity of *BnaMMS* requires validation via complementation or gene editing approaches. Furthermore, whether the A6 locus interacts with the known loci or other regulatory networks, and whether there is potential crosstalk with WUS-CLV components under specific developmental or environmental cues, warrants further investigation. Additionally, the agronomic implications of *BnaMMS*, such as its effects on yield, flowering time, and stress adaptation should be evaluated in diverse genetic backgrounds and field environments.

In conclusion, our study not only identified a novel QTL on chromosome A6 controlling MMS formation in rapeseed but also provided evidence for its likely involvement in auxin-related developmental pathways, distinct from the classical WUS-CLV regulatory axis. The trait’s significant potential for direct yield improvement and plant architecture optimization further enhances its value as a strategic genetic resource. These findings enrich the genetic resources available for stem architecture breeding and deepen our understanding of the complex and multifaceted regulatory mechanisms underlying stem determinacy in *Brassica* species.

## Conclusion

In this study, we identified and characterized a stable MMS germplasm in *Brassica napus*. Genetic analysis demonstrated that the MMS trait is controlled by a novel gene *BnaMMS*. Through BSA-seq and fine mapping, we narrowed *BnaMMS* to a 70-kb interval containing 17 annotated genes. Transcriptomic analysis of NILs revealed that only two of these genes were differentially expressed between MMS and SMS plants. Moreover, KEGG and GO enrichment analyses showed that the DEGs are mainly involved in auxin biosynthesis, tryptophan metabolism, and auxin transport. These findings suggest that *BnaMMS* likely induces the multi-stem phenotype by perturbing auxin homeostasis and distribution within the SAM. Our study not only provides a valuable genetic resource for plant architecture breeding in rapeseed but also offers novel insights into the molecular mechanisms underlying stem development.

## Supplementary Information

Supplementary Material 1.

Supplementary Material 2.

## Data Availability

DNA and RNA sequencing data were submitted to the NCBI Sequence Read Archive (SRA) under BioProject accession number PRJNA1452662.

## Abbreviations

MMS: Multi-main-stem
SMS: Single-main-stem
SNP: Single nucleotide polymorphism
Indel: Insertion / deletion
NIL: Near-isogenic line
SAM: Shoot apical meristem
RNA-seq: Ribonucleic acid sequencing
BSA-seq: Bulked segregant analysis sequencing
QTL: Quantitative trait loci
GO: Gene ontology
KEGG: Kyoto encyclopedia of genes and genomes
DH: Doubled haploid
DEG: Differentially expressed gene
ED: Euclidean distance
CI: Confidence interval
