# Supplementary material for: Fine mapping and transcriptomic analysis reveal BnaMMS, a novel locus regulating multi-main-stem phenotype via auxin pathway in rapeseed
Source: BMC Plant Biol. 2026 Jun 4;26:1344. doi: 10.1186/s12870-026-09172-8 (PMC13455139; doi:10.1186/s12870-026-09172-8)
Supplement: Supplementary file 1 [file 12870_2026_9172_MOESM1_ESM.docx]

**Supplementary Figures**

**Fig. S1. Identification of candidate interval for *BnaMMS*.** The distribution of SNP frequency differences between the MMS (M1F) and SMS (N1F) bulk pools was analyzed using three distinct algorithms: (A) the SNP-index method, (B) the Euclidean Distance (ED) algorithm, and (C) the G’ statistical approach. (D) A composite scatter plot visualizes the genome-wide distribution of SNPs based on the pooled sequencing data. The congruent peaks identified by all three methods delineate the candidate genomic region harboring the *BnaMMS* locus.

**Fig. S2. Development and validation of polymorphic markers in the F_2_ population.** (A) Screening for polymorphic markers between parental lines and bulked pools. (B) Validation of the newly developed polymorphic markers in a small F_2_ population. Lane 1-11, individuals from F_2_ population that exhibited SMI; lane12, 195FM; lane 13-23, individuals from F_2_ population that exhibited MMI; lane24, 1030R.

**Fig. S3. Sequencing depth of the parental line and bulked pool.**

**Fig. S4. Genome-wide distribution of SNPs between the parental lines.**

**Fig. S5. Summary of genetic variations between the parental lines.** Statistical analysis of the number of SNPs (A) and Indels (B) in different genomic regions. Statistical analysis of the mutation types resulting from SNPs (C) and Indels (D).

**Fig. S6. Heatmap of DEGs compared between the parental lines.**

**Fig. S7. Heatmap of upregulated DEGs associated with auxin metabolic process.**
